# O Método e o Mantra

**DOI:** 10.36660/abc.20201013

**Published:** 2021-03-03

**Affiliations:** 1 Hospital SOS Cardio FlorianópolisSC Brasil Hospital SOS Cardio, Florianópolis, SC - Brasil; 2 Massachusetts General Hospital Department of Anesthesia, Critical Care and Pain Medicine Harvard Medical School BostonMassachusetts EUA Harvard Medical School - Department of Anesthesia, Critical Care and Pain Medicine, Massachusetts General Hospital, Boston, Massachusetts - EUA

**Keywords:** COVID-19, Pandemia, Faculdades de Medicina, Ciências da Saúde, Tecnologia e Inovação, Educação Médica, Doutorado

## Introdução

Na época de minha residência em Cardiologia, um paciente optou por se tornar vegetariano após implantar uma válvula cardíaca metálica. Até aquele dia, ele anunciava orgulhoso que só comia carne, arroz e feijão. Ele passou muitos anos sem comer uma folha de alface, mas como um amigo sugerira que esse tipo de dieta aumentava o risco de calcificação de outras valvas cardíacas, optou pela mudança antes mesmo de checar a veracidade da informação. Ele havia sido avisado de que uma dieta rica em vegetais poderia dificultar o manuseio da anticoagulação, mas não teve dúvida: encontrara motivação para viver uma vida mais saudável depois da cirurgia pela qual passou.

Altivo, eliminou todos os tipos de carnes e passou a consumir apenas vegetais. Sentia-se muito melhor e já não tinha mais falta de ar. Convicto dos benefícios do seu novo estilo de vida, faleceu seis meses depois da cirurgia por conta de uma hemorragia intracraniana, já que passou a tomar mais anticoagulantes para compensar a dieta. Nosso paciente acertou no mantra, mas errou no método. Ele optou por uma vida supostamente mais saudável, mas colocada em prática de maneira indevida, na hora errada e não foi capaz de usufruir de sua disciplina e de seu novo estilo de vida.

O mantra representa uma convicção pessoal profunda, resumida em uma frase, palavra ou atitude que se repete e visa alcançar um estado de comunhão consigo mesmo e com o meio em que vivemos. Já o método é um processo organizado, uma técnica ou uma maneira de fazer algo de acordo com um plano lógico, com a intenção de atingir um objetivo previamente definido. Ele deve ser claro o bastante para ser reproduzido. O mantra é menos tangível, mais conceitual.

Método e mantra podem ser responsabilizados pelo sucesso ou fracasso de inúmeras iniciativas. Mais frequentemente que o mantra, o método costuma ser questionado e de modo geral, devemos assumir que o método precisa ser revisto quando um objetivo não foi alcançado. Discutimos menos o mantra por ele estar fundamentado em axiomas. No entanto, mantra e método são inseparáveis.

### Desencontros na pandemia

Num momento de completa irracionalidade em relação à pandemia da covid-19, que se caracteriza pelo desencontro de opiniões, condutas e a busca por uma solução redentora, além do triste número de fatalidades,^1^ vale a pena indagar sobre o que deu errado. Mantra ou método? Ou estariam, ambos, equivocados?

Ainda que apresentando efeitos que vão muito além das questões sanitárias, a pandemia é essencialmente um problema médico. Todavia, a incapacidade e o despreparo dos sistemas de saúde em propor soluções efetivas ficou evidente em todo o mundo. Todo o treinamento, conhecimento e experiência com situações historicamente semelhantes parece ter sido esquecido ou aparenta ser insuficiente e inadequado para lidar com a situação atual.

Não sou capaz de provar, mas se um plano de ação coordenado tivesse sido implementado, melhores resultados teriam sido obtidos, menos vidas seriam perdidas e estaríamos com menos sequelas. O desastre poderia ter sido ainda maior se não dispuséssemos da dedicação quase que instintiva dos abnegados profissionais de saúde, esses os verdadeiros heróis nessa confusão.

Na crise financeira de 2009, um plano coeso e a ação rápida e coordenada dos bancos centrais minimizou a crise. O problema foi sanado pelo conjunto dos técnicos em finanças. O que está dando errado na pandemia, então? Porque os sistemas de saúde e suas lideranças não conseguem propor uma ação ordenada? Mantra e método parecem irremediavelmente desconectados aqui.

### Mantras recorrentes, métodos propostos e suas consequências

Há tempos, escutamos três mantras recorrentes relativos à prática médica e à medicina no Brasil. Para cada mantra, um método foi proposto. O primeiro sugere que o país seja carente de médicos e, portanto, mais profissionais são imprescindíveis. Problema resolvido: inúmeras faculdades de Medicina foram abertas em todo o país.

O segundo mantra foca na necessidade de aperfeiçoamento acadêmico dos médicos: mais ciência para melhor servir. Em resposta, no ano de 1983 foi criado o TCC e a pós-graduação apresentou um substancial crescimento.

Por último, o mais recente dos mantras recomenda complementar a formação médica com cursos de aprimoramento em negócio para facilitar a comunicação entre médicos e administradores. Médicos e gestores juntos, falando a mesma língua – marketing, finanças, recursos humanos, contabilidade – inevitavelmente aperfeiçoariam a assistência. Consequentemente, nunca tantos médicos cursaram *Master in Business Administration* (MBA) como nos últimos anos.

As três vertentes poderiam ter convergido para um bem maior: mais médicos com melhor preparo acadêmico, sintonizados e trabalhando em conjunto com gestores! Mantra e método em consonância. Entretanto, o cenário que vemos é bem diferente do que foi imaginado. Parece-me que ocorreu o oposto: mais médicos com treinamento inadequado, sem nenhuma aptidão ao método científico e incapazes de entender o rigor da pesquisa, trabalhando, não em parceria, mas sob o comando dos gestores de saúde. Mantra e método em dissonância.

Tentaremos avaliar como essas variáveis podem ter separadamente ou em conjunto influenciado e resultado na completa desarticulação que ocorre continuamente durante a pandemia. Estas são peculiaridades da realidade brasileira e que não explicam a pífia capacidade dos sistemas de saúde de muitos países (mas não em todos) em reagir. Talvez estejamos, inclusive, usando a pandemia como pretexto para essa reflexão e seremos acusados de oportunismo ao tentar debater assuntos não necessariamente conectados a ela, mas vamos aos fatos.

### Número inadequado de médicos no Brasil

O Brasil tem cerca de 502.475 médicos, quase que simetricamente divididos entre homens e mulheres: 2,4 médicos para cada 1.000 habitantes,^2^ o que parece um número bastante razoável. Para tanto, em um país com 211,8 milhões de habitantes, temos 346 faculdades de medicina, 141 públicas e 205 privadas. Os Estados Unidos possuem 317,6 milhões de habitantes e 184 escolas médicas; a China tem 1,3 bilhão de habitantes e 158 escolas médicas. No Brasil, nos últimos cinco anos, formamos, em média, 30.000 novos médicos a cada 12 meses. São 10,4 médicos para cada 100 mil habitantes por ano, número distante de países como a Dinamarca que, em 2020, tinha 23 graduandos para cada 100 mil habitantes.

Aparentemente, os números não conversam entre si, dada a enorme discrepância. Isso acontece porque não deve existir um “número mágico”, tudo depende da estrutura dos sistemas de saúde em cada país. Entretanto, existe um coeficiente mínimo que compromete a qualidade da assistência e outro acima do qual ela não melhora. Esse número varia entre países, regiões e sistemas de saúde.

Não pretendemos determinar aqui qual seja o número adequado para o Brasil, mas supomos que seja de 2,5 médicos para cada 1.000 habitantes. Assumimos que este número se refira a médicos bem formados, mas essa informação não é conhecida ou sequer discutida. Como em todas as profissões, o serviço de dez profissionais mal qualificados pode ser feito por um bem qualificado. Portanto, de nada adianta a relação médico/habitante ser adequada se a qualidade do profissional não for.

Certo ou errado, dada a competição acirrada pelas vagas, o sistema de acesso aos cursos de Medicina continua a selecionar um grupo de jovens talentosos que acabam subaproveitados por falta de oportunidade dentro das universidades. Em muitas delas, não existe, por exemplo, a proximidade entre escolas de Medicina e hospitais para oferecer ao estudante o treinamento necessário. Em outras, não existem programas de residência médica (parte fundamental do treinamento médico) suficientes para acomodar todos os egressos.

A causa desse mau desempenho, portanto, não parece ser culpa dos alunos, mas da péssima qualidade de muitas faculdades, que não estão preparadas para a complexa tarefa de formar um médico em seis anos. Além disso, o Brasil sempre será carente de médicos enquanto for obrigação exclusiva destes trocar uma receita, preencher uma solicitação de exame ou fazer uma avaliação de pré-natal, um teste ergométrico ou um ultrassom de abdome. Jamais alcançaremos o número ideal de profissionais enquanto o “ato médico” – criado pelos próprios profissionais – continuar a justificar a abertura não planejada de cursos de Medicina por todo país. O mantra e o método precisam, portanto, mudar. Esquecer a obsessão pelo número – assumindo que todos os problemas podem ser resolvidos se houver profissionais suficientes – e focar na melhoria da qualidade para, então, redimensionar o tamanho da força de trabalho.

### Incentivo à pesquisa

Diante desse quadro desfavorável, foi criado um subterfúgio, ao meu ver incompreensível, chamado Trabalho de Conclusão de Curso (TCC). Ele não faz parte das Diretrizes Curriculares Nacionais para o curso de Medicina, mas vem sendo utilizado em faculdades privadas (e em algumas públicas) como regra institucional local. Em tese, trata-se de um trabalho acadêmico obrigatório e instrumento de avaliação final de um curso superior, elaborado em forma de dissertação, visando a iniciação e envolvimento do aluno com a pesquisa científica. Qual teria sido a premissa dessa exigência que não seja aludir que a realização de pesquisa ou de um trabalho científico melhora a qualificação do medico?

Não posso afirmar que acontece em outras disciplinas, mas julgo que nada pode ser mais equivocado do que aplicar esse conceito à Medicina. São inúmeros os exemplos de médicos de imenso talento que não possuem aptidão para pesquisa, bem como de excelentes pesquisadores que não se sentem confortáveis em interagir com pacientes. Ocasionalmente, os dois interesses podem convergir em um mesmo profissional sem que isso o torne necessariamente melhor em quaisquer das frentes. A excelência clínica não depende de excelência em pesquisa. Médicos podem e devem aprender a interpretar artigos científicos sem que para isso precisem realizar tais pesquisas, o que seria vital para evitar que em crises como a atual, posturas anticiência fossem adotadas. Quem sabe não teríamos que passar pelo constrangimento de disputar a importância e o papel de ensaios clínicos randomizados com estudos observacionais.

Nesse sentido, o TCC pouco ajuda: os médicos que gostam de pesquisar não precisam dele para continuar fazendo. Os médicos que não gostam se sentem usados e o interesse, que já era pequeno, desaparece. O TCC deveria ser opcional e os melhores trabalhos devidamente reconhecidos, promovidos e premiados com o intuito de inspirar outros alunos.

A exigência do TCC contrasta frontalmente com a relativa facilidade de aceitação para participar em um doutorado em Medicina. Em alguns programas, a tese de doutorado será o primeiro e único trabalho apresentado pelo autor. Muitas vezes, o trabalho e a bolsa de pesquisa a ele associada servem exclusivamente para manter a relação do médico com a instituição hospitalar e não para alcançar a finalidade primordial do doutoramento: o avanço do conhecimento em uma área especifica, através da produção de conhecimento inédito e de alto nível, feito por alguém que busca aprofundamento científico e/ou acadêmico sob a orientação de pessoas e grupos que dotam de profundo conhecimento em suas áreas de estudo. O doutorado deveria representar o ápice da carreira e não uma finalidade em si. Por isso, não deve ser utilizado como instrumento de incentivo à pesquisa.

Além disso, poucas universidades brasileiras adotam o sistema de compilação da obra do autor dentro de uma determinada linha de pesquisa. Posso citar como exemplo uma abordagem diferente para o doutoramento, acontecida recentemente, em que um dos autores participou de duas bancas de médicos australianos. O trabalho foi enviado por *e-mail*: um compêndio de mais de 500 páginas reunindo várias contribuições do autor (trabalhos inéditos publicados em revistas cientificas, com revisores independentes) sobre o tema abordado e um resumo final conectando todas as observações coletadas em uma conclusão relevante. Dessa forma, o avaliador não interage com o aluno, o que mantém a impessoalidade e a concentração no valor do trabalho, que são próprias nessa circunstância. As considerações do avaliador são enviadas para a universidade, que decide se o aluno merece ou não o título.

Tal experiência contrasta com a de algumas universidades nacionais nas quais, por vezes, amigos e familiares participam da apresentação do trabalho de doutoramento sem que este e seu autor(a) tenham passado pelo crivo cientifico adequado, o que, em tese, seria o papel da aula de qualificação. É comum, entretanto, que o time escalado para a qualificação não tenha o peso dos membros da banca final. O ideal seria exatamente o oposto: as maiores autoridades no tema deveriam formar a banca da aula de qualificação e os jovens pesquisadores, como uma maneira de prestigiálos, avaliar a apresentação final da tese. Insistimos em atuar ao contrario, porque existe uma expectativa de validação do processo, antes que tenha sido realmente validado. Talvez por isso exista um enorme interesse na obtenção de um título de PhD em Medicina no Brasil. Em outros países, médicos de grande proeminência acadêmica não possuem doutorado e ou se interessam pelo titulo. Mais importante do que a titulação em si é a contribuição para o conhecimento.

A qualidade da pesquisa, entretanto, agregada ou não a programas de pós-graduação, é fundamental para avaliar hospitais e instituições de ensino. Invariavelmente, o naipe da instituição está associado à realização de pesquisa: quanto mais se pesquisa, melhor a universidade. Por isso, grandes hospitais incentivam o convívio entre médicos e pesquisadores. No Brasil, a equação se mostra mais complicada, já que a maioria da produção cientifica médica nacional é realizada em faculdades públicas ou em hospitais ligados a estas faculdades que, a rigor, contam com menos recursos e não conseguem oferecer o mesmo nível de atendimento dos hospitais privados, nos quais a produção acadêmica é pequena. Desse modo, não há estímulo para a pesquisa em hospitais e faculdades desta categoria. Obviamente existem exceções, mas esta é a regra e torcemos para que estas inspirem o planejamento estratégico das demais. O mantra é apropriado, mas o método é equivocado: há uma completa inversão de valores no que diz respeito ao estímulo à pesquisa no Brasil.

### MBA para médicos

Para complicar ainda mais toda essa conjuntura, outro desencontro nos parece evidente: muitos médicos, ao longo da carreira, legitimamente se interessam em aprender sobre gestão; inúmeras oportunidades de aperfeiçoamento estão disponíveis nesta área. Vários profissionais são convidados a fazer tais cursos, algumas vezes oferecidos pelo próprio hospital em que exercem as suas atividades. Isso parece válido, mas talvez uma abordagem mais razoável e equilibrada fosse possível: para cada médico cursando MBA, um gestor seria matriculado em um curso de mesma duração para aprender e compreender o fundamento do pensamento médico.

Por enquanto, o mantra insiste em dizer que os médicos precisam compreender melhor a intrincada relação comercial envolvida na prestação do serviço. Somos médicos, não provedores. Não temos clientes, temos pacientes. A perspectiva de médicos e gestores é completamente diferente, pois a decisão em relação ao indivíduo, à sua saúde e à sua vida é sempre mais complexa. Muitos gestores não conhecem esse ponto de vista: o grupo é impessoal, ao contrário do indivíduo. O erro ou acerto do gestor impacta a sua bonificação. A decisão do médico traz outras consequências.

Embora pareça razoável sugerir ao médico que aprenda a linguagem do gestor para melhorar a qualidade e eficiência do serviço prestado, a hipótese inversa não pode ser afastada. Infelizmente, a maioria dos médicos-gestores invariavelmente assumem o papel de representante dos gestores frente ao grupo de médicos, nunca ao contrário. Ao invés de inovar, se transformam em meros zeladores de processos pré-existentes e a colaboração é interrompida.

### O que fazer?

Vamos precisar redesenhar o mantra e o método para compatibilizar os interesses entre médicos, pacientes, população e gestores. O conceito geral é simples: melhores escolas medicas, que incentivem o talento natural de cada aluno, relacionadas ou não à pesquisa e que criem uma identidade profissional que nos permita interagir em igualdade com outros atores envolvidos na prestação de serviço médico, centrados em instituições que valorizem a pesquisa visando nortear seu planejamento estratégico e que, por essa razão, se tornem referência. Dessa forma, talvez tenhamos melhor sorte na próxima pandemia.
