# Supplementary material for: O Método e o Mantra
Source: Arq Bras Cardiol. 2021 Mar 3;116(3):367–70. [Article in Portuguese] doi: 10.36660/abc.20201013 (PMC8159571; doi:10.36660/abc.20201013)
Supplement: Translation

# Translation: The Method and the Mantra

**Affiliations:** 1 FlorianópolisSC Brazil Hospital SOS Cardio, Florianópolis, SC - Brazil; 2 BostonMassachusetts USA Harvard Medical School - Department of Anesthesia, Critical Care and Pain Medicine, Massachusetts General Hospital, Boston, Massachusetts - USA

**Keywords:** COVID-19, Pandemics, Schools Medical, Health Sciences, Technology and Innovation Management, Medical Education, Doctor’s Degree

## Introduction

At the time of the residency in Cardiology, a patient chose to become a vegetarian after having a metallic heart valve implanted. Until that day, he proudly announced that he only ate meat, rice and beans! He had spent years without eating a lettuce leaf, but as a friend suggested that this type of diet increased the risk of calcification of the other heart valves, he chose to change it, even before checking the veracity of the information.

He had been warned that a diet rich in vegetables could make it difficult to handle anticoagulation, but he had no doubts: he had found the motivation to live a healthier life after the surgery. He proudly eliminated meats and chose vegetables, instead. He felt much better and he no longer had shortness of breath. Convinced of the benefits of his new lifestyle, he died 6 months after surgery due to intracranial hemorrhage, as he had to take more anticoagulants to compensate for the diet… Our patient got the mantra right, but not the method. He opted for a supposedly healthier life that was wrongly put into practice, at the wrong time and was unable to enjoy his discipline or the new lifestyle.

A mantra represents a deep conviction, summed up in a phrase, word or attitude that is repeated in order to achieve a state of communion with oneself and the environment where we live. The method is an organized process, technique or way of doing something, according to a logical plan, aiming to achieve a previously defined objective. The method must be clear enough to be reproduced. The mantra is less tangible, more conceptual. Method and mantra can be responsible for the success or failure of innumerous initiatives. More often than the mantra, the method is often questioned and it is generally assumed that the method needs to be revised, when an objective cannot be achieved. Less frequently, the mantra is discussed because it is based on axioms. But the mantra and the method are inseparable.

### Disagreements in the Pandemic

In a moment of complete irrationality regarding the COVID-19 pandemic, which, in addition to the sad number of fatalities, is characterized by a disparity of opinions, conduct and a search for a redeeming solution,^1^ it is worth asking about what went wrong: mantra or method? Or were both, method and mantra, wrong?

Even with effects that go far beyond health issues, the pandemic is essentially a medical problem. However, the inability and unpreparedness of health systems to propose effective solutions has been evident throughout the world. It is as if all the training, knowledge and experience with historically similar situations, had been forgotten or was insufficient and inadequate to deal with the current situation. I cannot prove it, but if a coordinated action plan had been implemented, better results would have been obtained, fewer lives would have been lost and there would have been fewer sequelae. If not for the almost instinctive dedication of selfless health professionals, who are the real heroes in this whole mess, the disaster could have been even greater.

During the 2009 financial crisis, a cohesive plan and rapid and coordinated action by the central banks minimized the crisis. It was a financial problem that was solved by the group of financial experts. What is going wrong in the pandemic, then? Why are health systems and their leaders unable to propose an orderly action? Mantra and method seem hopelessly disconnected here.

### Recurring Mantras, Proposed Methods and Their Consequences:

We have been long listening to three recurring mantras related to medical practice and Medicine in Brazil. For each mantra, a method has been proposed. The first one suggests that the country needs doctors and, therefore, more and more doctors are essential. Problem solved: several medical schools were opened throughout the country.

The second mantra focuses on the doctors’ need for academic improvement: more science to better serve! In response to that, the ‘Final Term Paper’ was created in 1983 and postgraduate school grew.

Finally, the most recent of the mantras, recommends complementing medical training with business improvement courses to facilitate the communication between doctors and administrators. Doctors and managers together, speaking the same language – marketing, finance, human resources, accounting – would inevitably improve care. Therefore, never have so many doctors attended a Master in Business Administration (MBA) course as in recent years.

In principle, the three components could have converged to the greater good: more doctors with better academic training, tuned in and working side by side with managers! Mantra and method in harmony. However, the scenario we see is quite different from the one we imagined. It seems like the opposite has happened: many more doctors with inadequate training, without any aptitude for the scientific method and unable to understand the stringency of research, working not in partnership, but under the control of health managers. Mantra and method in dissonance.

We will try to assess how these variables may have influenced, either separately or together, resulting in the complete disarticulation that continues to occur during the pandemic. Of course, these are peculiarities of the Brazilian reality and do not explain the meager capacity of health systems to react in many countries (not all). Perhaps, we are even using the pandemic as a pretext for this reflection and we might be accused of opportunism when trying to debate issues not necessarily connected to it; however, let us get to the facts.

### Inadequate number of physicians in Brazil

Brazil has approximately 450,000 doctors almost symmetrically divided between men and women: 2.2 doctors per 1,000 inhabitants.^2^ It seems a pretty reasonable number. Therefore, in a country with around 200 million inhabitants, we have 350 medical schools, 105 public and 245 private ones. The United States has 300 million people and 131 medical schools; China has 1.3 billion people and 150 medical schools. In Brazil, we have trained 30,000 new doctors every year for at least 5 years. There are 15 doctors per 100,000 inhabitants per year, a number far from that found in countries such as Denmark, which in 2015 had 23 medical graduates per 100,000 inhabitants.

Apparently, the numbers do not talk to each other, given the huge discrepancy. This is because there seems to be no magical number. It all depends on how health systems are structured in each country. It is obvious, however, that there is a number below which the quality of care is compromised, but another one above which the quality does not improve. And that number varies between countries, health regions and systems. We do not intend to determine herein what the appropriate number for Brazil is, but it is supposed to be 2.5 doctors per 1,000 inhabitants. It is thought that this number refers to well-trained doctors but this information is neither known nor discussed. However, as in all professions, the work of 10 poorly-qualified professionals can be done by a single well-qualified one. Therefore, it is of no use if the doctor/inhabitant ratio is adequate, if the quality of the professional is not.

Right or wrong, given the tremendous competition for vacancies, the system of access to medical courses continues to select a group of talented young people who end up being underutilized for lack of opportunity inside the medical schools. In many of them, for instance, there is no proximity between medical schools and hospitals to provide the student with the necessary training. In others, there are not enough medical residency programs to accommodate all medical graduates. And the residency is a vital part of the medical training. The cause of this poor performance, therefore, does not seem to be the students’ fault, but the poor quality of training in many medical schools, which are not ready for the complex task of training a doctor in 6 years.

Moreover, Brazil will always be in need of physicians as long as it is the exclusive obligation of a physician to change a prescription, fill out an exam request, make a prenatal assessment, perform an exercise test or an abdominal ultrasound. We will never reach the ideal number of professionals as long as the “medical act” – created by doctors themselves – continues to justify the unplanned opening of medical schools across the country. The mantra and the method, therefore, have to change. Forget the obsession with numbers – assuming that everything can be solved if there are enough people – and focus on improving quality so that the size of the workforce can be resized.

### Research Incentive

Amid this hubbub, a subterfuge was created, which is incomprehensible from my viewpoint, called ‘Final Term Paper’ (FTP). The FTP is not part of the National Curricular Guidelines for the Medical Course but has been used in private and some public colleges, as a local institutional rule. In theory, it is a mandatory academic work and an instrument for the final evaluation of a higher education course, prepared in the form of a dissertation, aiming at the student’s initiation and involvement with scientific research. What would the premise of this requirement have been if it did not suggest that conducting research or scientific work improves the medical doctor’s qualification?

I do not know about other disciplines, but there is nothing more wrong than applying this concept to Medicine. There are countless examples of very talented doctors who have no aptitude for research, as well as excellent researchers who do not feel comfortable interacting with patients. Occasionally, the two interests can converge on the same professional, without making it necessarily better on either side. Clinical excellence does not depend on research excellence. Doctors can and must learn to interpret scientific articles without having to carry out such research. This would indeed be vital, to avoid that in crises such as the current one, unscientific postures be adopted. Who knows, we would not have to go through the embarrassing situation of disputing the importance and role of randomized clinical trials compared to observational studies. In this sense, the FTP is of little help: those who like research do not need it to continue researching. Those who do not like research, feel used and their interest, which was already scarce, disappears. The FTP should be optional, and the best papers should be duly recognized, promoted and awarded to inspire other students.

The FTP requirement contrasts head-on with the relatively easy acceptance to participate in a doctorate in Medicine. In some programs, the doctoral thesis will be the first and only work submitted by the author. Very often, the work and the research grant associated to it, is used exclusively to maintain the doctor’s relationship with the hospital and not to achieve the primary purpose of getting a PhD: the advancement of knowledge in a specific area, through the production of unprecedented and high-level knowledge, made by someone who seeks scientific and / or academic growth under the guidance of people and groups who have deep knowledge in those areas of study. The doctorate should represent the apex of the career and not an end in itself. Therefore, it cannot be used as an instrument to encourage research.

Moreover, few Brazilian universities use the system for compilation of the author’s work inside a given line of research. As an example of a different approach to the doctorate, last year, one of the authors participated in two doctorate Board of Examiners of Australian doctors. The work was sent by e-mail: a compendium of more than 500 pages, bringing together several contributions by the author (unprecedented works published in scientific journals with independent reviewers) on the addressed topic and a final summary connecting all these observations into a relevant conclusion. Therefore, the evaluator does not interact with the student, maintaining the impersonality and concentrating on the value of the work, which are proper under this circumstance. The evaluator’s considerations are sent to the university, which decides whether the student deserves the Ph.D. title.

This experience contrasts with some of the national ones where, sometimes, friends and family participate in the presentation of the doctoral work, without that work and its author going through the appropriate scientific screening. In theory, this would be the role of the qualification class. Commonly, however, the team selected for the examination does not have the impact of the members of the final board. Ideally, it would be exactly the opposite: the board that comprises the examination class, including the greatest authorities on the subject and young researchers, aiming to accolade them, should evaluate the final thesis presentation. We insist on doing the opposite, because there is an expectation of validating the process, before it has actually been validated. Perhaps that is why there is a huge interest in obtaining a Ph.D. degree in Medicine in Brazil. In other countries, physicians of great academic prominence do not have a doctorate and are not interested in having the title. Much more important than the degree itself is the contribution to knowledge.

The quality of research, however, whether or not added to postgraduate programs, is essential to assess hospitals and educational institutions. Invariably, the institution’s category is associated with the performance of research: the more research, the better the institution. For this reason, large hospitals encourage the interaction between doctors and researchers. In Brazil, the equation becomes more complicated, since the vast majority of the national medical scientific production is carried out in public colleges or in hospitals linked to these colleges, which, as a rule, have fewer resources and are unable to offer the same level of care to patients as private hospitals do, where there is little academic production. Therefore, there is no incentive for research in private hospitals and colleges. Evidently, there are exceptions, but this is the rule. One hopes these exceptions will serve as inspiration for the strategic planning of others. Appropriate mantra, but wrong method: there is a complete inversion of values in relation to the stimulation of research in Brazil.

### MBA for Medical Doctors

To further complicate this entire situation, another disparity seems evident to us. Many physicians, throughout their careers, are legitimately interested in learning about management. Several opportunities for improvement are available in this area. Many professionals are invited to take such courses, sometimes offered by the hospital itself, where they concentrate their activities. All of this seems valid, but perhaps a more reasonable and balanced approach would be possible: for each physician taking an MBA, a manager would be enrolled in a course of the same duration to learn how to understand the foundation of medical thinking.

For the time being, the mantra insists that we doctors need to better understand the intricate business relationship involved in providing the service. But we are physicians, not service providers. We do not have customers; we have patients. The perspectives of doctors and managers are completely different ones, because the decision regarding the individual, their health, and their life, is always more complex. Many managers do not know this point of view: the group is impersonal, the individual is not. The manager’s failure or success impacts his bonus. The doctor’s decision has other consequences. Although it seems reasonable to suggest that physicians should learn the managers’ language to improve the quality and efficiency of the service provided by them, the inverse hypothesis cannot be ignored. Unfortunately, most medical managers invariably assume the role of managers’ representative to the group of doctors; never the other way around. Instead of innovating, they become mere caretakers of pre-existing processes and the collaboration gets stalled.

### What should be done?

We must redesign the mantra and the method to reconcile the interests between doctors, patients, population and managers. The general concept is a simple one: better medical schools, encouraging each student’s natural talent, whether or not related to research, creating a professional identity that allows us to interact on an equal basis with other actors involved in the provision of medical service, centered on institutions that value research to guide their strategic planning and, for that reason, become a reference. Perhaps by doing this, we will have better luck in the next pandemic.
